# Changes in air quality and human mobility in the USA during the COVID-19 pandemic

**DOI:** 10.1007/s42865-020-00019-0

**Published:** 2020-10-26

**Authors:** Cristina L. Archer, Guido Cervone, Maryam Golbazi, Nicolas Al Fahel, Carolynne Hultquist

**Affiliations:** 1 College of Earth, Ocean, and Environment, University of Delaware, Newark, Delaware 19716 USA; 2 Institute for Computational and Data Science, The Pennsylvania State University, University Park, Pennsylvania, 16802 USA; 3 Biden School of Public Policy and Administration, University of Delaware, Newark, 19716 Delaware USA; 4 Center for International Earth Science Information Network (CIESiN), The Earth Institute at Columbia University, Palisades, New York, 10964 USA

**Keywords:** COVID19, Air pollution, Nitrogen dioxide, Particulate matter, Mobility, Social distancing, Pandemic

## Abstract

The first goal of this study is to quantify the magnitude and spatial variability of air quality changes in the USA during the COVID-19 pandemic. We focus on two pollutants that are federally regulated, nitrogen dioxide (NO_2_) and fine particulate matter (PM_2.5_). NO_2_ and PM_2.5_ are both primary and secondary pollutants, meaning that they can be emitted either directly into the atmosphere or indirectly from chemical reactions of emitted precursors. NO_2_ is emitted during fuel combustion by all motor vehicles and airplanes. PM_2.5_ is emitted by airplanes and, among motor vehicles, mostly by diesel vehicles, such as commercial heavy-duty diesel trucks. Both PM_2.5_ and NO_2_ are also emitted by fossil-fuel power plants, although PM_2.5_ almost exclusively by coal power plants. Observed concentrations at all available ground monitoring sites (240 and 480 for NO_2_ and PM_2.5_, respectively) were compared between April 2020, the month during which the majority of US states had introduced some measure of social distancing (e.g., business and school closures, shelter-in-place, quarantine), and April of the prior 5 years, 2015–2019, as the baseline. Large, statistically significant decreases in NO_2_ concentrations were found at more than 65% of the monitoring sites, with an average drop of 2 parts per billion (ppb) when compared to the mean of the previous 5 years. The same patterns are confirmed by satellite-derived NO_2_ column totals from NASA OMI, which showed an average drop in 2020 by 13% over the entire country when compared to the mean of the previous 5 years. PM_2.5_ concentrations from the ground monitoring sites, however, were not significantly lower in 2020 than those in the past 5 years and were more likely to be higher than lower in April 2020 when compared with those in the previous 5 years. After correcting for the decreasing multi-annual concentration trends, the net effect of COVID-19 at the ground stations in April 2020 was a reduction in NO_2_ concentrations by − 1.3ppb and a slight increase in PM_2.5_ concentrations by + 0.28 μg/m^3^. The second goal of this study is to explain the different responses of these two pollutants, i.e., NO_2_ was significantly reduced but PM_2.5_ was nearly unaffected, during the COVID-19 pandemic. The hypothesis put forward is that the shelter-in-place measures affected people’s driving patterns most dramatically, thus passenger vehicle NO_2_ emissions were reduced. Commercial vehicles (generally diesel) and electricity demand for all purposes remained relatively unchanged, thus PM_2.5_ concentrations did not drop significantly. To establish a correlation between the observed NO_2_ changes and the extent to which people were actually sheltering in place, thus driving less, we used a mobility index, which was produced and made public by Descartes Labs. This mobility index aggregates cell phone usage at the county level to capture changes in human movement over time. We found a strong correlation between the observed decreases in NO_2_ concentrations and decreases in human mobility, with over 4 ppb decreases in the monthly average where mobility was reduced to near 0 and around 1 ppb decrease where mobility was reduced to 20% of normal or less. By contrast, no discernible pattern was detected between mobility and PM_2.5_ concentrations changes, suggesting that decreases in personal-vehicle traffic alone may not be effective at reducing PM_2.5_ pollution.

## Introduction

The World Health Organization (WHO) estimates that about 91% of the world population is exposed to poor air quality and that 4.2 million people die each year from causes directly attributed to air pollution (World Heath Organization (WHO) 2020b). Nitrogen dioxide (NO_2_) is one of a group of highly reactive gases known as nitrogen oxides (NO_*x*_). NO_2_ can irritate the human respiratory system and is also harmful to ecosystems by the formation of nitric acid and acid rain (U.S. Environmental Protection Agency (EPA) 2020a; Lin and McElroy 2011). NO_2_ is also a precursor to tropospheric ozone (O_3_) formation, which has further negative impacts on human health (EPA 2020b). PM_2.5_ is another harmful air pollutant that consists of microscopic particles with a diameter smaller than 2.5 μm. These particles can pose a great risk to human health because they can penetrate into human lungs and even the bloodstream; PM_2.5_ is also often associated with poor visibility (EPA 2020c). NO_2_ and PM_2.5_ are both primary (i.e., they can be directly emitted into the atmosphere) and secondary (i.e., they can also form after chemical reactions in the atmosphere) pollutants. High concentrations of both are not necessarily found where their emissions are highest, due to processes such as chemical reactions, transport, or diffusion. NO_2_ and PM_2.5_ are the main focus of this paper because they are among the seven “criteria” pollutants that are regulated at the federal level by the EPA via the National Ambient Air Quality Standards (NAAQS).

The novel coronavirus disease (COVID-19 hereafter) was first identified in Wuhan, China, on December 30, 2019 (WHO 2020a; Chan et al. 2020). Cases started to spread initially in China but quickly expanded to other countries across the world. COVID-19 was declared a global pandemic on March 11, 2020 (WHO Regional Office for Europe 2020). At the time of this study, over 36 million people have been affected by the virus, with over 1 million deaths in 214 countries and territories (Worldometers.info 2020; Johns Hopkins University 2020). COVID-19 first reached the USA in January 2020 and since then it has caused over 212,000 deaths (Center for Disease Control and Prevention (CDC) 2020; Johns Hopkins University 2020). The death rate of COVID-19 is significantly higher among people with cardiovascular and respiratory illnesses (Mullen 2020), which is also strongly connected with air pollution (Isaifan 2020). Furthermore, new studies suggest that higher concentrations of air pollutants result in a higher risk of COVID-19 infection (Yongjian et al. 2020) and mortality (see Conticini et al. (2020) and pre-print by Wu et al. (2020)). Conticini et al. (2020) provided evidence that the high level of pollution in Northern Italy, especially the Lombardia and Emilia Romagna regions, was an additional co-factor to explain the high level of lethality recorded in that area. After adjusting for 20 potential confounding factors, such as population size, age distribution, and population density, Wu et al. (2020) found, with strong statistical confidence, that “a small increase in long-term exposure to PM2.5 leads to a large increase in the COVID-19 death rate.” By contrast, Contini and Costabile (2020) caution that the exposure to air pollution alone could not explain the spread and mortality of COVID-19. Citing the high mortality rates in northern Italy, Spain, the USA, and the UK, which are characterized by very different pollution levels, and the lack of significant outbreaks in very highly populated and polluted cities in India, they suggest that several other factors are potentially involved in the spread of COVID-19 and its mortality, such as population density, social habits, the restrictive measures applied, meteorological conditions, and the different strategies used for counting deaths related to COVID-19 and infected people. Clearly, the link between air pollution and spread or mortality of COVID-19 is still under debate.

In the USA, social distancing measures were implemented state by state with the goal of limiting the spread of the pandemic. In general, closure or non-physical interaction options (e.g., delivery only) were implemented for schools, restaurants, and public places of gathering. Businesses, workers, and types of activities that were deemed essential during the pandemic either continued operating under strict protection measures (e.g., personal protective equipment (PPE), masks) or switched to online. Non-essential businesses requiring physical presence and interaction closed completely (e.g., hair salons, bars, gyms). The extent of social distancing measures, seriousness of the implementation, and the degree of compliance varied throughout the USA. Most states announced some level of social distancing orders starting in mid-March 2020 (British Broadcasting Corporation (BBC) 2020), often including a mandatory quarantine for people diagnosed with or showing likely COVID-19 symptoms. By the beginning of April, almost all states had a mandatory shelter-in-place or lockdown order (Mervosh et al. 2020). Hereafter, lockdown and shelter-in-place will be used interchangeably. The social distancing measures have led to drastic changes in mobility and energy use and therefore changes in emissions of pollutants.

Globally, the COVID-19 outbreak is forcing large changes in economic activities (Worden et al. 2020). In China, following the strict social distancing measures, transportation decreased noticeably and, as a result, China experienced a drastic decrease in atmospheric pollution, specifically CO (Worden et al. 2020), NO_2_, and PM_2.5_ (Zambrano-Monserrate et al. 2020; Worden et al. 2020; National Aeronautics and Space Administration (NASA) 2020) concentrations in major urban areas. However, emissions from residential heating and industry remained steady or slightly declined (Chen et al. 2020). Using satellite data, Zhang et al. (2020) and the National Center for Atmospheric Research (Worden et al. 2020) reported a 70% and 50% decrease in NO_*x*_ concentrations in Eastern China, respectively. Bao and Zhang (2020) showed an average of 7.8% decrease in the Air Quality Index over 44 cities in northern China. Bauwens et al. (2020) and Shi and Brasseur (2020) reported an increase in O_3_ concentrations in the same region. Chen et al. (2020), reported that NO_2_ and PM_2.5_ concentrations were decreased by 12.9 and 18.9 μg/m^3^, respectively. They estimated that this improvement in the air quality of China avoided over 10,000 NO_2_- and PM_2.5_-related deaths during the pandemic, which could potentially outnumber the confirmed deaths related to COVID-19 in China (Chen et al. 2020). Other researchers also have proposed that the improvements of air quality during the pandemic might have saved more lives than the COVID-19 has taken (Dutheil et al. 2020; Burke 2020). Likewise, Isaifan (2020) argues that the shutting down of industrial and anthropogenic activities caused by COVID-19 in China may have saved more lives by preventing air pollution than by preventing infection.

European countries, such as France and Italy, experienced a sharp reduction in their air pollution amid COVID-19 (European Space Agency (ESA) 2020). In Brazil, a significant decrease in CO concentrations and, to a lesser extent, in NO_*x*_ levels was observed, while ozone levels were higher due to a decrease in NO_*x*_ concentrations in VOC-limited locations (Dantas et al. 2020; Nakada and Urban 2020). The same findings were observed in Kazakhstan and Spain, respectively (Kerimray et al. 2020; Tobías et al. 2020). Likewise, Iran (Nemati et al. 2020) and India (Mahato et al. 2020; Sharma et al. 2020) reported noticeable improvements in air pollution during the pandemic. Le Quéré et al. (2020) looked into the impacts of the forced confinement on CO_2_ emissions and concluded that global CO_2_ emissions decreased by 17% by early April compared with the average level in 2019. They predict that the yearly mean CO_2_ emissions would decrease by 7% if restrictions remain until the end of 2020.

In the USA, as a result of social distancing, states started to experience a dramatic decrease in personal transportation and mobility in general (Gao et al. 2020). Personal vehicle transportation decreased by approximately 46% on average nationwide, while freight movement only decreased by approximately 13% (Pishue 2020). Air traffic decreased significantly as well (Slotnick 2020). On-road vehicle transportation is a major source of NO_*x*_ emissions (EPA 2017) while airports are usually hot spots for NO_2_ pollution (NASA 2020).

The Houston Advanced Research Center (HARC) (Beydoun et al. 2020) analyzed the daily averages of hourly aggregated concentrations of benzene, toluene, ethylbenzene, and xylenes (BTEX) across six stations in Houston, USA. They reported a decrease in BTEX levels in the atmosphere while there was an intensified formation on PM_2.5_ in the region. Similarly, the New York Times reported huge declines in pollution over major metropolitan areas, including Los Angeles, Seattle, New York, Chicago, and Atlanta using satellite data (Plumer and Popovich 2020).

While a noticeable number of studies have looked into the correlation between lockdown measures amid COVID-19 and air quality in different countries, none has evaluated air quality for the entire USA. The goals of this study are to investigate the magnitude and spatial variability of air quality (NO_2_ and PM_2.5_) changes in the USA during the COVID-19 pandemic and to understand the relationships between mobility and NO_2_ changes. An innovative aspect of this study is that we use an extensive database of ground monitoring stations for NO_2_ and PM_2.5_ (Section 2.1) and a third-party high-resolution mobility dataset derived from cellular device movement (Section 2.3). In addition, we included satellite-retrieved NO_2_ information to increase the spatial data coverage (Section 2.2). Whereas most studies rely only on a comparison to 2019, we consider five prior years (2015–2019) to provide a more robust measure of changes in pollution level across the country amid the COVID-19 pandemic.

## Data

### Air quality data

Criteria pollutant concentration data, originally measured and quality-checked by the various state agencies, are centrally collected and made available to the public by the EPA through their online Air Quality System (AQS or AirData) platform (EPA Air Quality System 2020). For NO_2_ and PM_2.5_, the reported concentrations are 1-h averages, thus 24 records are reported daily (if no records are missing). The AQS pre-generated files are updated twice per year: once in June, to capture the complete data for the prior year, and once in December, to capture the data for the summer. The daily files, containing daily average and daily maximum of 1-h NO_2_ concentrations and 24-h average of PM_2.5_ concentrations, were downloaded for the years 2015–2019. At the time of this study (May 2020), however, the pre-generated files for April 2020 were not yet available.

For the year 2020 only, the data source was the U.S. EPA AirNow program (EPA AirNow 2020), which collects real-time observations of criteria pollutants from over 2000 monitoring sites operated by more than 120 local, state, tribal, provincial, and federal agencies in the USA, Canada, and Mexico. As stated on AirNow website, “these data are not fully verified or validated and should be considered preliminary and subject to change.” Of the two types of files available from AirNow, namely AQObsHourly and Hourly, AQObsHourly files were downloaded for March and April 2020 because of their smaller file size (they are updated once per hour, as opposed to multiple times). Texas and New York do not feed NO_2_ measurements to Airnow, thus their 2020 NO_2_ data were downloaded directly from their state websites (Texas Commission on Environmental Quality (TCEQ) 2020; New York State Department of Environmental Conservation 2020).

The NAAQS for NO_2_ and PM_2.5_ are based on the comparison of a “design value,” which is a specific statistic of measured concentrations over a specific time interval, against a threshold value as follows:

- NO_2_: annual mean of 1-h concentrations may not exceed 53 parts per billion (ppb);
- NO_2_: 98th percentile of 1-h daily maximum concentrations, averaged over 3 years, may not exceed 100 ppb;
- PM_2.5_: annual mean of 24-h concentrations, averaged over 3 years, may not exceed 12 μg/m^3^;
- PM_2.5_: 98th percentile of 24-h concentrations, averaged over 3 years, may not exceed 35 μg/m^3^.

Clearly, it is not possible to calculate the design values as early as April because neither the annual average nor the 98th percentile can be calculated after only 4 months. As such, in this study, we will use a simple monthly average as the representative metric to compare the concentrations in April 2020 to those in the previous five Aprils.

An air quality station, whether measuring NO_2_ or PM_2.5_, was used in this study only if it reported both in 2020 (through AirNow) and in the five years prior (through AQS). In addition, only air quality stations that were reporting at least 75% of the time were retained. Note that not all NO_2_-measuring sites also measure PM_2.5_, and vice versa. Of the 426 and 882 sites that measured NO_2_ and PM_2.5_, respectively, in April 2020, only 271 and 819 reported at least 75% of the time, and ultimately only 201 and 480 reported NO_2_ and PM_2.5_ also in April 2015–2019 for at least 75% of the time. These are the sites that we will focus on in this study and that are shown in Figs. 5 and 6.

### Satellite data

Satellite observations for NO_2_ were acquired using the OMI instrument flying onboard the NASA Aura satellite (Krotkov et al. 2019) and were downloaded using the NASA GIOVANNI portal (NASA 2020). Specifically, the Nitrogen Dioxide Product (OMNO2d) was used, which is a Level-3 global, daily, gridded product at a 0.25^∘^× 0.25^∘^ spatial resolution provided for all pixels where cloud fraction is less than 30% (Krotkov et al. 2019). The product comes in two variants, the first measuring the concentration in the total column and the second the concentration only in the tropospheric portion of the column. For this work, the latter measurements were used. The Aura satellite is not geostationary, but polar-orbiting, which means that it orbits over a given location at about the same time each day. Therefore, changes in NO_2_ column totals due to COVID-19 that occur at other times of the day may not be well captured by the OMI retrievals. Details about the NASA OMI measurements can be found in the literature (Boersma et al. 2011).

**Fig. 1:**
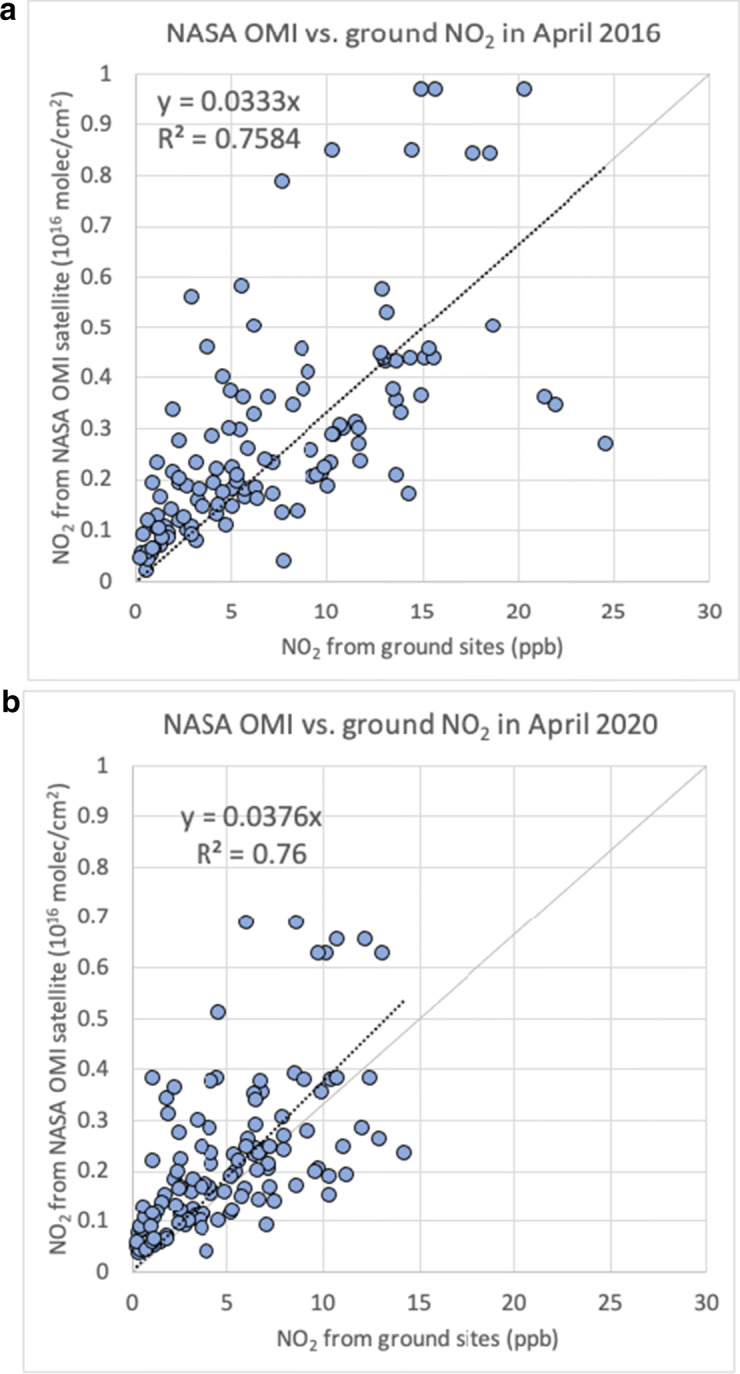
Scatter plots of monthly-average NO_2_ concentrations (ppb) from the ground monitoring sites versus monthly-average NO_2_ column totals (10^16^ molecules/cm^2^) retrieved from the NASA OMI satellite at the pixels of the ground monitoring sites during **a** April 2016 and **b** April 2020

The satellite-derived NO_2_ column totals at the pixels of the ground monitoring sites are well correlated with the NO_2_ concentrations recorded at the ground monitoring sites in all years, with R-square values varying between 0.76 and 0.80. As an example, we show the correlation between the two in 2016 and 2020 in Fig. 1. Similar agreements between satellite-derived NO_2_ and observations or modeling results have been reported in the literature (Valin et al. 2011; Russell et al. 2012). As such, we can use satellite-derived NO_2_ column totals to (1) confirm the results obtained from the ground monitoring sites and (2) analyze pixels where no ground monitoring sites are available.

### Mobility data

Mobility measures aim to capture general patterns of observed movement and most available data products today utilize mobile device activity as a proxy. While policy makers set social distancing guidance, there are various policies enacted and various degrees to which policies are followed throughout the country. We seek to observe actual patterns of movement by using a dataset developed by Descartes Labs (2020b) that provides an aggregated mobility measure based on anonymized and/or de-identified mobile device locations. Mobility is essentially a statistical representation of the distance a typical member of a given population moves in a day. Descartes Labs calculated the farthest distance apart recorded by smartphone devices utilizing select apps (with location reporting enabled) for at least 10 uses a day, spread out over at least 8 h in a day, with a day defined as 00:00 to 23:59 local time (Warren and Skillman 2020). The maximum distance for each qualifying device is tied to the origin county in which the anonymized user is first active each day. Aggregated results at the county level are produced as a statistical measure of general travel behavior.

Mobility data are ultimately provided as percent of normal, i.e., the ratio of aggregated mobility during each day of the COVID-19 pandemic over that of the baseline (17 February–7 March 2020). Note that the baseline period is in late winter 2020, whereas the period of focus in this paper is April 2020, in spring. As such, a fraction of the differences in mobility may be due to differences in weather and/or climate rather than to COVID-19 restrictions. We did not attempt to correct for this type of bias. Another caveat, noted by the producers of the data (Warren and Skillman 2020), is that the raw data used to calculate mobility are available for only a small fraction of the total number of devices (a few percent at most), thus the resulting mobility may or may not truly represent the average behavior in each county. Nonetheless, the effects of these sampling errors are expected to be small. The mobility data are made freely available by Descartes Labs at the US county level (Descartes Labs 2020a).

## Results

### Observed air quality changes

In the rest of this paper, we will compare the monthly average of the pollutant of interest—NO_2_ or PM_2.5_—during the month of April 2020 to the average of the five monthly averages during April of the years 2015 through 2019. There are two reasons for this choice. First, using five years to establish a reference is more meaningful than, say, using just the year 2019, because year-to-year variability can occur regardless of the pandemic. In fact we found that, in general, the year 2019 was relatively clean when compared to the previous five, thus a comparison between April 2020 and April 2019 may underestimate the true impact of COVID-19. Second, although the monthly average is not the design value for either NO_2_ or PM_2.5_, it is a value that is representative of the overall air quality during the entire month of April. Alternative metrics, such as the monthly maximum, are more representative of extreme circumstances, like wildfires, that are not necessarily associated with COVID-19.

**Fig. 2:**
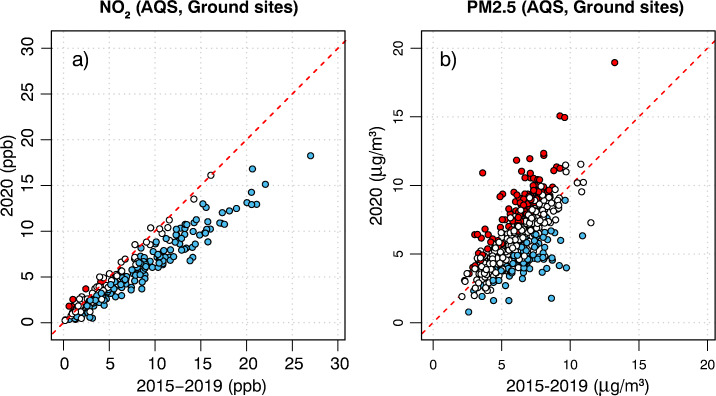
Scatter plots of **a** NO_2_ and **b** PM_2.5_ observed concentrations at the AQS ground monitoring sites during April of 2020 (y-axis) versus April of the previous five years 2015–2019 (x-axis). Blue-filled markers represent sites for which the values in April 2020 were lower than any in April of 2015–2019; red-filled markers represent sites for which the values in April 2020 were higher than any in April of 2015–2019

Starting with NO_2_, the April 2020 averages were generally below the April 2015–2019 average at the ground monitoring sites, as most sites lay below the 1:1 line in Fig. 2a. In addition, 65% of the sites were characterized by NO_2_ concentrations in 2020 that were lower than those in all of the previous five years (for the month of April). Only a few sites (5 in total, < 2%) experienced NO_2_ concentrations in 2020 that were higher than those in all of the previous five years (for the month of April). The average drop in NO_2_ concentrations in April 2020 with respect to the average of the previous five years was − 2.02 ppb (Tables 1 and 3). To verify that the observed reduction was not simply due to the multi-annual decreasing trend in emissions (Jiang et al. 2018), a trend analysis was performed. The average slope of the linear fit was negative (− 0.24 ppb/year) and, after de-trending, the net contribution of COVID-19 was approximately − 1.3 ppb on average.

**Table 1:** Monthly average of NO_2_ concentrations (ppb) by state in the month of April of the years 2015–2020 from the AQS sites. The last column shows the percent change in 2020 with respect to the 2015–2019 mean

State	No. sites	2015	2016	2017	2018	2019	2020	
Arizona	4	12.49	11.68	13.58	12.82	10.33	9.06	− 25.6%
California	56	9.73	9.23	8.79	8.85	8.13	5.92	− 33.8%
Colorado	7	11.37	9.87	9.39	9.94	9.5	8.25	− 17.6%
Connecticut	3	11.71	11.13	11.72	10.34	8.43	6.54	− 38.7%
District of Columbia	1	9.19	8.74	7.43	8.25	8.46	7.26	− 13.7%
Florida	7	5.65	4.23	4.73	5.55	5.34	4.77	− 6.5%
Georgia	2	11.39	12.15	10.83	11.62	10.8	10.83	− 4.7%
Hawaii	1	3.04	2.99	4.59	3.21	3.72	2.85	− 18.8%
Indiana	4	11.49	9.43	8.32	9.89	8.2	7.2	− 23.9%
Iowa	1	1.42	2.33	1.66	2.08	1.9	1.69	− 10.0%
Kansas	4	6.10	4.86	4.4	5.98	5.16	4.47	− 15.6%
Kentucky	1	13.94	14.95	13.08	13.18	17.43	11.25	− 22.5%
Maine	2	4.30	3.67	3.99	4.03	3.39	2.83	− 27.0%
Maryland	5	10.43	10	8.57	8.84	8.11	6.74	− 26.7%
Massachusetts	8	9.47	9.08	7.15	8.45	6.47	5.15	− 36.6%
Michigan	2	9.37	8.28	8.37	8.59	7.39	6.79	− 19.2%
Minnesota	2	7.51	5.95	7.25	9.88	6.53	5.08	− 31.6%
Mississippi	1	3.91	4.25	4.28	3.85	3.79	2.68	− 33.3%
Missouri	6	10.81	9.17	8.82	8.62	8.64	6.92	− 24.9%
Montana	1	0.54	0.61	0.9	0.68	0.46	0.36	− 43.5%
Nevada	2	8.48	7.93	9.77	10.09	8.53	6.76	− 24.5%
New Jersey	8	13.80	13.39	12.33	12.48	12.38	8.03	− 37.6%
New Mexico	8	4.68	4.56	4.48	4.74	5.22	3.58	− 24.4%
New York	5	13.64	12.56	12.05	12.42	11.3	7.55	− 39.1%
North Carolina	3	6.04	6.29	6.05	6.11	6.58	4.4	− 29.1%
North Dakota	6	2.91	1.98	2.44	2.59	2.16	1.95	− 19.3%
Ohio	5	14.60	12.13	10.84	11.17	11.28	8.25	− 31.3%
Oklahoma	3	8.89	8.43	7.02	7.38	7.38	6.02	− 23.0%
Oregon	2	12.39	11.59	10.69	9.42	9.98	7.77	− 28.2%
Pennsylvania	1	12.87	12.89	11.74	10.81	8.7	10.39	− 8.9%
Rhode Island	2	14.03	13.5	10.23	13.37	10.15	8.47	− 30.9%
South Carolina	2	5.59	6.03	6.73	6.62	5.84	4.92	− 20.2%
Texas	40	5.80	6.23	5.23	6.41	5.95	5.26	− 11.2%
Utah	7	3.42	2.95	4.5	4.15	2.92	2.32	− 35.3%
Vermont	2	7.32	6.12	6.23	6.16	5.74	4.00	− 36.7%
Virginia	9	5.77	5.05	4.92	5.25	5.42	4.04	− 23.5%
Washington	2	16.80	18.17	14.25	13.32	11.36	9.52	− 35.6%
Wisconsin	2	12.50	11.11	9.61	11.36	9.75	8.63	− 20.6%
Wyoming	13	1.69	1.62	1.61	1.55	1.31	1.33	− 14.5%

**Fig. 3:**
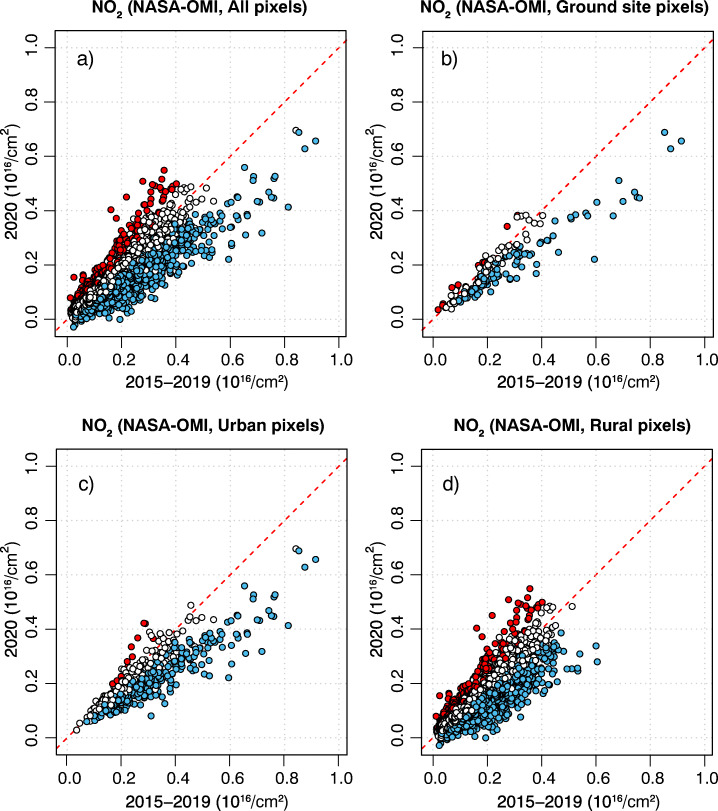
Scatter plots of NO_2_ column totals from NASA-OMI satellite at **a** all pixels over the continental US (CONUS), **b** at the pixels of the AQS ground stations, **c** at the urban pixels over CONUS, and **d** at the rural pixels over CONUS, during April of 2020 (y-axis) versus April of the previous five years 2015–2019 (x-axis). Blue-filled markers represent sites for which the values in April 2020 were lower than any in April of 2015–2019; red-filled markers represent sites for which the values in April 2020 were higher than any in April of 2015–2019

The same pattern is confirmed in the satellite-derived data. Out of the 227 pixels with ground monitoring sites, a total of 127 (56%) exhibited lower NO_2_ in 2020 than in the previous five years and only 5% higher (Fig. 3b). Once all 14,706 pixels with valid satellite retrievals over the continental US are considered, a similar pattern of lower NO_2_ column totals in 2020 than in the five previous years emerges from these data too (Fig. 3a), but with 28% of the pixels lower in 2020 than in the previous five years and 5% higher (for the month of April, Table 3).

**Fig. 4:**
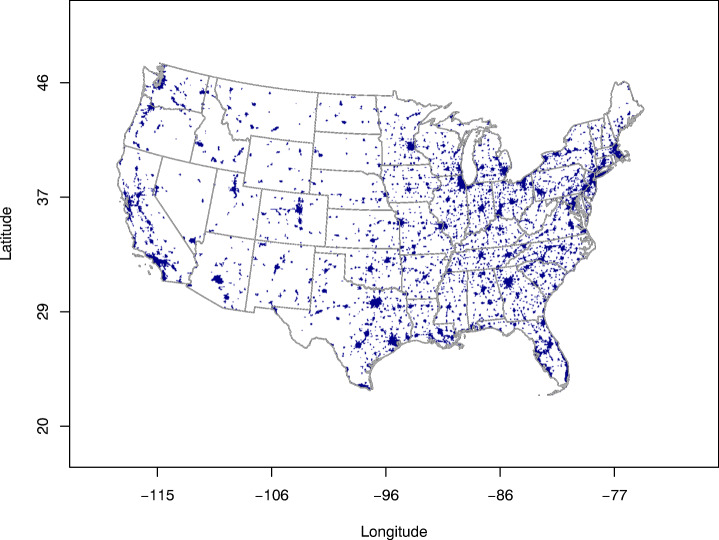
Map of the urban pixels in the continental US from the Socioeconomic Data and Applications Center (SEDAC) (SEDAC 2020)

Further insight was gained from the satellite-derived NO_2_ column totals by differentiating the pixels into two categories: urban versus rural (Socioeconomic Data and Applications Center (SEDAC) 2020). The urban pixels are shown in Fig. 4. For urban pixels, the findings closely resemble those from the AQS ground monitoring sites, with less than 2% of the sites (11 out of 786) experiencing higher NO_2_ in April 2020 than in the previous five years and over 40% of the sites (325 out of 786) experiencing the opposite (Fig. 3c). This is not surprising, as ground monitoring sites are more likely to be placed in urban locations, where most people live. Basically all the pixels with higher NO_2_ in April 2020 than in the previous five years from Fig. 3a were rural pixels (Fig. 3d).

**Fig. 5:**
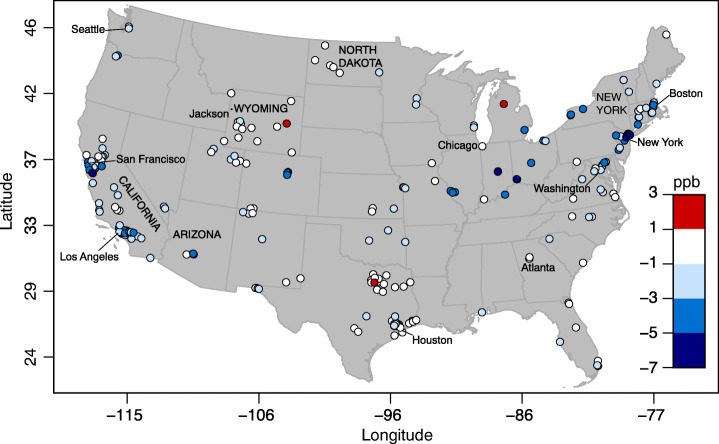
Difference in monthly average NO_2_ concentrations (ppb) between April 2020 and the five previous Aprils (2015–2019). Negative values (blue) indicate a decrease in NO_2_ concentrations in April 2020, vice versa positive values (red) indicate an increase. Latitude and longitude are in degrees. Cities and states mentioned in the paper are labelled in lower and upper cases, respectively

In terms of spatial variability, Fig. 5 shows that, although NO_2_ reductions were recorded all over the country, the highest decreases were observed in California and the Northeast, where the shelter-in-place measures started earlier (March 11 for California, the earliest in the country, and March 22 for New York, third earliest (Mervosh et al. 2020)) and lasted longer (both states still have major restrictions in place as of June 10, 2020 (The Washington Post 2020)). Noticeable exceptions were North Dakota and Wyoming, where either no significant decreases or actual small increases in NO_2_ concentrations were observed. North Dakota enforced no shelter-in-place measures and in Wyoming only the city of Jackson implemented a stay-at-home order as of April 20, 2020 (Mervosh et al. 2020). However, as discussed in Section 2.3, people’s actual mobility, as opposed to state ordinances, is a more appropriate metric to capture the effect of COVID-19 on air quality because there was no complete compliance with state or city restrictions.

**Fig. 6:**
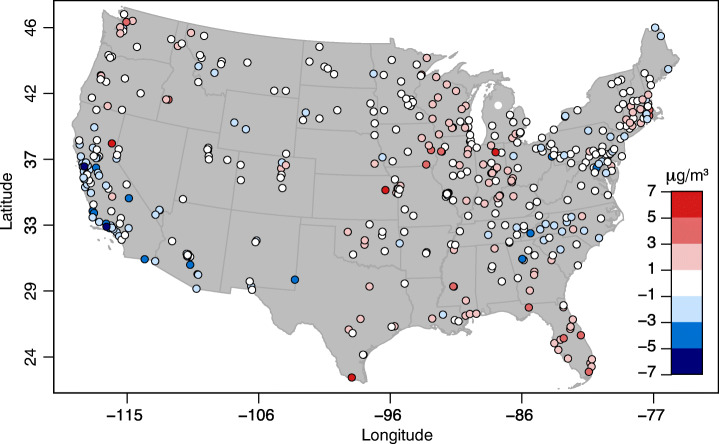
Difference in monthly-average PM_2.5_ concentrations (μg/m^3^) between April 2020 and the five previous Aprils (2015–2019). Negative values (blue) indicate a decrease in PM_2.5_ concentrations in April 2020, vice versa positive values (red) indicate an increase. Latitude and longitude are in degrees

**Fig. 7:**
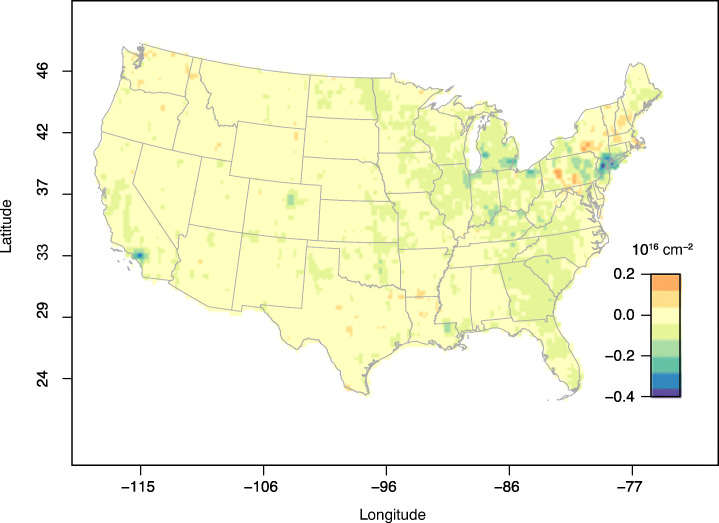
Difference between the NASA OMI NO_2_ monthly average column totals (10^16^ molecules/cm^2^) in 2020 and in 2015–2019 for the month of April. The “hotspot” of reduced NO_2_ in the Northeast is apparent. Latitude and longitude are in degrees

Figure 5 was useful because it included actual NO_2_ concentrations measured near the ground. However, the spatial coverage was sparse and urban areas were over-sampled compared with rural areas. This weakness is addressed via the NASA OMI satellite data, which are shown in Fig. 7 as the difference between the monthly average of NO_2_ column total in 2020 and that in 2015–2019 for the month of April. The regions with low coverage of ground concentration of NO_2_ and mobility in the Midwest are generally characterized by near-normal NO_2_ column totals. The Northeast hotspot of low mobility is also a hotspot of low NO_2_, consistent with Bauwens et al. (2020), although it is surrounded by patches of above-normal values that were not detectable from the ground monitoring stations. The Los Angeles area is another hotspot of NO_2_ decreases, as well as low mobility.

For PM_2.5_, the ground monitoring stations depict a completely different response to COVID-19 (Fig. 6). Whereas most NO_2_ sites were laying below the 1:1 line (Fig. 2a), the majority of PM_2.5_ sites laid above it (Fig. 2b), indicating an overall increase in monthly average PM_2.5_ in the country in April 2020 with respect to the previous five years. Only 18% of the sites reported concentrations of PM_2.5_ that were lower in 2020 than in the previous five years (in the month of April), while 24% of the stations reported the highest levels in 2020 compared to the previous five years (for the month of April). The average increase in PM_2.5_ concentrations with respect to the mean of the previous five years was small, + 0.05 μg/m^3^ (Tables 2 and 3). Results from the trend analysis indicated that, in the absence of COVID-19, the average concentration of PM_2.5_ in April 2020 would slightly decrease (slope − 0.12 μg/m^3^/year). Thus, the net contribution of COVID-19 on PM_2.5_ concentrations was a slight increase of about 0.28 μg/m^3^ on average.

**Table 2:** Monthly average of PM_2.5_ concentrations (μg/m^3^) by state in the month of April of the years 2015–2020 from the AQS sites. The last column shows the percent change in 2020 with respect to the 20152019 mean

State	No. sites	2015	2016	2017	2018	2019	2020	
Alabama	4	7.94	7.56	8.82	7.26	7.64	8.59	+ 9.5%
Alaska	4	3.45	3.57	4.36	3.43	4.45	3.83	− 0.6%
Arizona	13	5.67	5.82	6.9	8.13	5.03	4.75	− 24.7%
Arkansas	4	7.01	7.12	8.12	6.98	7.51	7.54	+ 2.6%
California	61	7.06	6.65	6.35	7.93	6.31	5.26	− 23.3%
Colorado	8	5.31	3.65	4.63	5.87	5.26	5.21	+ 5.4%
Connecticut	8	4.8	5.54	3.81	5.61	5.32	5.75	+ 14.6%
Delaware	3	5.73	5.65	6.21	6.48	5.91	7.09	+ 18.2%
District Of Columbia	1	6.21	6.68	7.11	8	6.33	4.67	− 32.0%
Florida	16	7.01	7	7.41	7.68	6.39	9.52	+ 34.1%
Georgia	10	7.87	8.31	8.52	8.25	8.83	8.44	+ 1.0%
Hawaii	7	5.96	4.72	6.68	4.26	3.2	3.32	− 33.1%
Idaho	5	4.47	5.51	4.21	4.43	3.55	5.72	+ 29.0%
Illinois	14	8.23	7.67	7.06	8.03	7.43	8.11	+ 5.5%
Indiana	15	7.5	8.21	6.17	6.64	6.21	8.47	+ 21.9%
Iowa	9	7.78	6.97	6.18	7.09	5.65	8.71	+ 29.3%
Kansas	3	6.34	6.21	6.85	8.31	8.77	11.47	+ 57.2%
Kentucky	12	6.97	7.03	6.27	6.48	6.77	8.04	+ 19.9%
Louisiana	4	7.81	7.06	8.43	7.52	6.84	7.74	+ 2.8%
Maine	6	5.16	5.64	4.93	4.46	3.63	4.05	− 15.0%
Maryland	10	6.93	6.75	5.74	6.46	4.33	4.81	− 20.4%
Massachusetts	9	4.67	5.08	3.02	5.6	4.68	5.71	+ 23.9%
Michigan	11	6.7	6.7	5.52	6.48	6.33	7.1	+ 11.9%
Minnesota	18	5.09	5.66	5.06	6.22	4.88	5.73	+ 6.5%
Mississippi	7	7.98	7.49	8.32	8.27	6.97	10.15	+ 30.0%
Missouri	13	7.65	6.3	6.46	7.33	7.33	6.6	− 5.9%
Montana	11	5.02	4.57	4.07	4.81	3.7	4.21	− 5.1%
Nebraska	2	8.02	6.65	9.82	9.77	6.1	8.17	+ 1.2%
Nevada	6	6.16	4.81	4.57	5.25	3.23	4.11	− 14.4%
New Hampshire	5	4.3	4.2	3.18	4.11	3.34	4.01	+ 4.8%
New Jersey	3	5.69	7.27	7.32	7.17	6.09	5.84	− 12.9%
New Mexico	5	7.34	5.05	7.02	7.93	5.58	4.87	− 26.0%
New York	7	5.32	4.99	4.38	4.93	4.92	4.5	− 8.3%
North Carolina	13	6.78	7.25	7.16	6.53	6.4	5.41	− 20.7%
North Dakota	6	4.65	3.24	4.33	5.26	3.58	4.2	− 0.3%
Ohio	12	7.49	7.86	5.55	6.82	6.68	6.99	+ 1.6%
Oklahoma	9	7.32	7.31	7.52	8.78	7.71	8.19	+ 6.0%
Oregon	12	5.3	4.94	4.28	5.14	4.24	5.17	+ 8.2%
Pennsylvania	24	7.41	7.01	7.64	6.99	6.52	6.97	− 2.0%
Rhode Island	5	4.84	5.2	4.56	6.01	3.3	4.08	− 14.7%
South Carolina	6	7.33	6.95	7.95	6.64	6.6	6.62	− 6.7%
South Dakota	8	6.65	4.93	5.35	5.54	3.68	5.01	− 4.2%
Tennessee	11	6.41	6.68	6.8	6.45	6.5	6.23	− 5.1%
Texas	11	10.04	8.84	9.13	8.79	8.76	10.27	+ 12.7%
Utah	7	5.6	3.3	3.47	4.59	3.03	3.79	− 5.2%
Vermont	4	4.32	4.24	3.27	4.69	4.48	4.73	+ 12.6%
Virginia	6	5.74	5.76	6.33	5.31	5.75	5.66	− 2.0%
Washington	11	5.57	6.5	3.53	4.01	4.17	5.11	+ 7.4%
Wisconsin	18	5.45	7.47	4.58	5.46	6.68	7.49	+ 26.3%
Wyoming	3	4.04	2.75	2.73	3.3	2.57	1.73	− 43.8%

In summary, we report a large decrease (− 2.02 ppb, or 27%) in monthly average NO_2_ concentrations across the US ground monitoring stations, confirmed by the satellite-derived NO_2_ column total decrease of 7.1 × 10^14^ molecules/cm^2^ (or 24%) at the pixels of the ground monitoring stations, during April of 2020 when compared with April of the previous five years. When all the pixels with valid data were included, a drop of 2.4× 10^14^ molecules/cm^2^ (or 13%) during April of 2020 was observed when compared to April of the previous five years (Table 3). After de-trending, the net effect of COVID-19 on NO_2_ concentrations at the AQS ground monitoring stations was − 1.3 ppb. The monthly average of PM_2.5_, however, increased slightly on average (+ 0.05 μg/m^3^ when compared with the previous 5-year average) during the same period (Table 3). After de-trending, the effect of COVID-19 was a net increase of PM_2.5_ concentrations at the AQS ground monitoring stations by + 0.28 μg/m^3^. In the next Section 3.3, we try to explain the reasons for these differences.

**Table 3:** Average air quality measurements in April of the years 2015–2020 from NASA OMI and ground monitoring stations

	2015	2016	2017	2018	2019	2020
NASA OMI						
NO_2_ at all pixels (10^16^molecules/cm^2^)	0.16	0.15	0.14	0.13	0.14	0.12
NO_2_ at ground sites (10^16^molecules/cm^2^)	0.32	0.31	0.28	0.29	0.29	0.23
Ground monitoring stations						
NO_2_ (ppb)	8.16	7.69	7.22	7.62	7.0	5.52
PM_2.5_ (μg/m^3^)	6.52	6.36	6.02	6.60	5.82	6.31

### Observed mobility changes

**Fig. 8:**
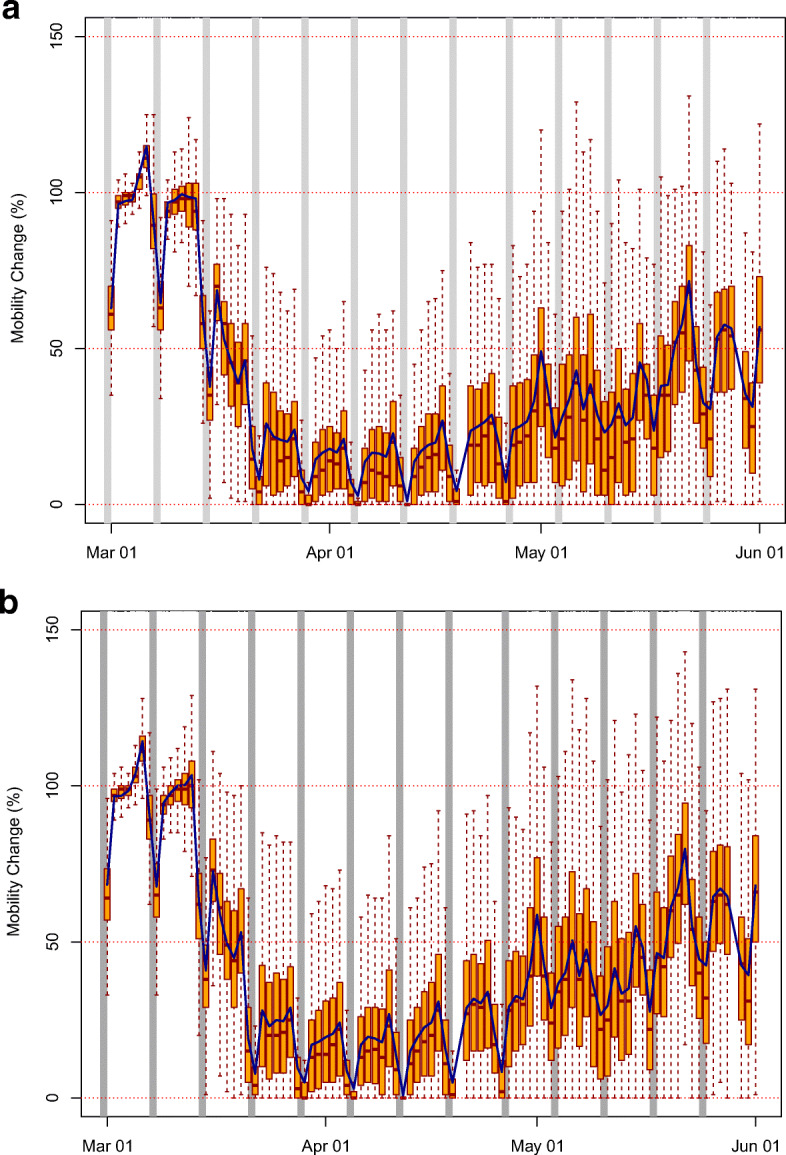
Mobility, expressed as percent of normal, at the locations of the AQS ground stations monitoring **a** NO_2_ and **b** PM_2.5_ during March–May 2020. The orange bars indicate first and third quantiles; the red-dashed lines cover the 5th to the 95th percentiles; the median is thick red solid; and the average of all the stations is shown with a blue line

Time series of mobility data at the counties with NO_2_ ground monitoring sites are shown in Fig. 8a and at the counties with PM_2.5_ ground monitoring sites in Fig. 8b. Only a few counties had both types of monitoring sites, thus the counties included in the two figures are generally different. Yet, the patterns are very similar. First of all, mobility on average dramatically dropped starting in the second half of March, reaching values around 20% by April, and then started to recover in May, as some states reopened for business or relaxed the shelter-in-place measures (The Washington Post 2020). Second, a distinct minimum in mobility during the month of April is clearly visible, which confirms that this month was the most relevant for air quality impacts from COVID-19. There is some variability around this general behavior, but nonetheless only a few counties barely reached normal mobility in April. Lastly, the typical traffic reduction during the weekends is confirmed in the mobility data, regardless of the pandemic. This adds confidence to the use of mobility data as a proxy for people’s actual behaviors.

**Fig. 9:**
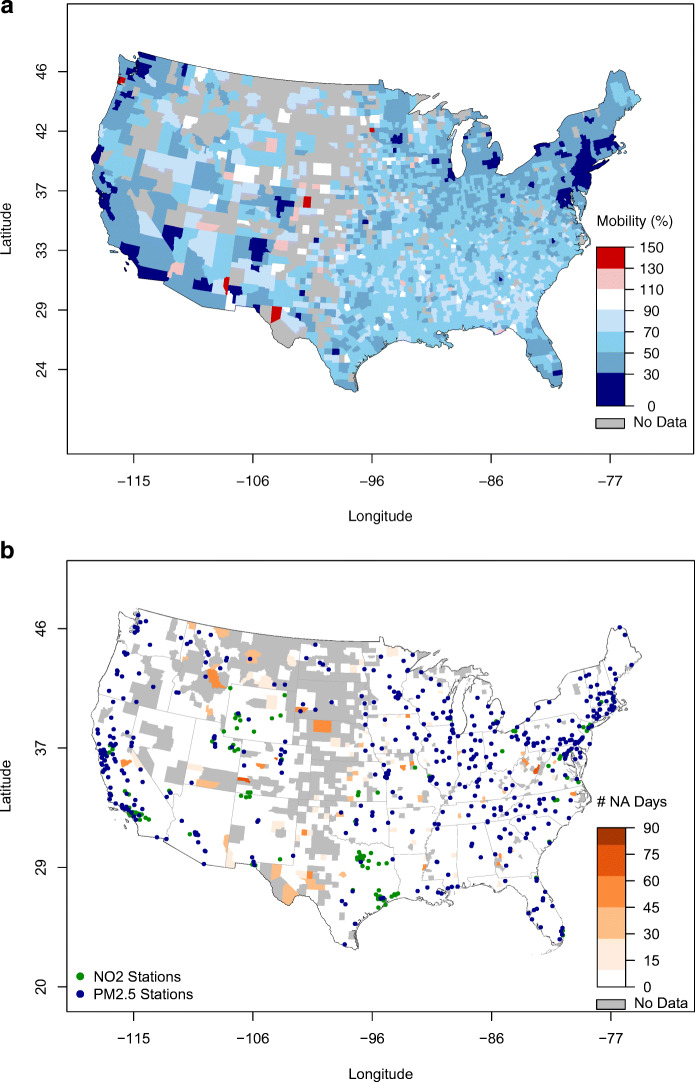
Spatial distribution of **a** mobility, expressed as percent of normal, in April 2020 and **b** mobility data availability, expressed as number of missing days, during March–May 2020. Latitude and longitude are in degrees

In terms of spatial variability, changes in mobility during COVID-19 in the USA were not uniform, although in general mobility was reduced in most states (Fig. 9a). Note the lack of data in many counties in the Midwest (in grey in Fig. 9b), possibly due to low population density, limited smartphone usage and cellular coverage. However, the ground monitoring stations of both NO_2_ and PM_2.5_ are generally located in counties with mobility data availability. In general, the strongest decreases in mobility are found around large urban areas throughout the country, e.g., the Northeast corridor from Washington D.C. to Boston; the San Francisco and Los Angeles areas in California; Seattle in the Northwest; and Chicago. A few isolated counties experienced increases in mobility (in red in Fig. 9a). Wyoming stands out as one of the few states with no significant decreases in mobility, consistent with the lack of shelter-in-place measures (Mervosh et al. 2020).

### Relationships between air quality and mobility changes

To better interpret the relationship between mobility and the air pollutant of interest, either NO_2_ or PM_2.5_, the mobility data were divided into bins, based on the monthly average (in April 2020) of the mobility in the county where each ground monitoring site was located. For most cases, there was only one ground monitoring site per county. But in some cases, such as Los Angeles county in California for NO_2_ or Maricopa county in Arizona for PM_2.5_, multiple monitoring sites were located in the same county and therefore they were all paired to the same mobility value. The change in monthly average concentration of the pollutant between April 2020 and the five previous Aprils was then calculated for each mobility bin.

**Fig. 10:**
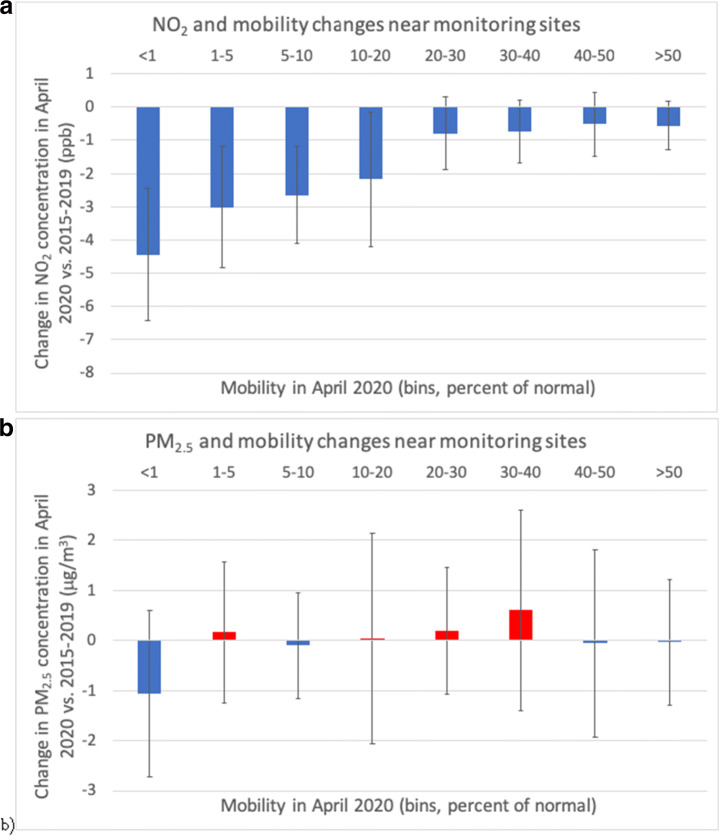
Changes in monthly average concentrations of **a** NO_2_ and **b** PM_2.5_ near ground monitoring sites during April of 2020 versus April of the previous five years as a function of mobility index bins in April 2020

Starting with NO_2_, there is a clear relationship with mobility (Fig. 10a). Large and negative changes in NO_2_ concentrations, on the order of − 4 ppb, were found at locations where mobility was basically halted, i.e., where it was less than 1% of normal in April 2020, as in full lockdown. As mobility increased, the NO_2_ benefits decreased, although not linearly. For example, decreases by 2–3 ppb in NO_2_ concentrations occurred where mobility was restricted but not to a full lockdown (i.e., between 1 and 20% of normal). Past 20%, the changes in NO_2_ concentrations were still negative and significant, but not large, less than 1 ppb on average. This suggests that NO_2_ responds modestly to changes in mobility that are not large, but then, if mobility is reduced dramatically (i.e., by at least 80%, thus it is down to 20% of normal), large decreases in NO_2_ can occur.

With respect to PM_2.5_, there is no obvious relationship between the reductions in mobility and the observed concentrations (Fig. 10b). Only for the most extreme mobility reductions, i.e., the bin with < 1% mobility, which indicates that the entire population was sheltering at home for the entire month of April, PM_2.5_ concentrations decreased by about 1 μg/m^3^. After the first bin, as mobility increased, both increasing and decreasing concentrations of PM_2.5_ were found, with large standard deviations and no discernible pattern. We conclude that the changes in PM_2.5_ were not directly caused by changes in people’s mobility.

How can we reconcile the clear relationship of NO_2_ with mobility with the lack thereof for PM_2.5_? The hypothesis we put forward is that the shelter-in-place measures mostly affected people’s driving patterns, thus passenger vehicle—mostly fueled by gasoline—emissions were reduced and so were the resulting concentrations of NO_2_. Commercial vehicles (generally diesel) and electricity demand for all purposes (often provided by coal-burning power plants), however, remained relatively unchanged; thus, PM_2.5_ concentrations did not drop significantly and did not correlate with the mobility index.

To test this hypothesis, in a subsequent study, we will use a photochemical model, coupled with a numerical weather prediction model, which we will run with and without emissions from diesel vehicles, while keeping everything else the same. The difference between the concentrations of the pollutants in the two cases will be attributable to diesel traffic alone. Similarly, we will be able to reduce emissions from other sectors, to reflect the effect of COVID-19 on other aspects of life, such as air traffic, business closures, or residential heating. We will explore the relationship between reductions of the mobility index and residential heating increases, as more people staying at home during lockdowns likely cause higher residential heating emissions, including from biomass burning.

## Conclusions and future work

This study analyzed the effects of COVID-19 on air quality, more specifically NO_2_ and fine particulate PM_2.5_ concentrations, in the USA. Although different states introduced different levels of shelter-in-place and social distancing measures at different times, by the beginning of April 2020 all states but a few had adopted some restrictions. As such, the analysis focused on the month of April 2020, which was compared to April of the previous five years, 2015–2019.

Two types of measurements were used, NO_2_ and PM_2.5_ concentrations from the ground monitoring stations—maintained by the states—and satellite-derived NO_2_ column totals in the troposphere. Although the two measurements are not identical, they are strongly correlated with one another because the near-ground concentrations of NO_2_ are the dominant contributors to the tropospheric column total.

To quantify social distancing, we used the mobility index calculated and distributed by Descartes Labs. Their algorithms account for people’s maximum distance travelled in a day by tracing the user’s location multiple times a day while using selected apps. Mobility is represented as a percent value, such that 100% means normal conditions, i.e., those during the period of 17 February–7 March 2020.

We found that NO_2_ levels decreased significantly in April 2020 when compared with April of the five previous years, by up to 8 ppb in the monthly average at some locations. On average over all US monitoring sites, the decrease in NO_2_ levels was between 24% (from satellite) and 27% (from ground stations). The decreases in NO_2_ were largest where mobility was reduced the most, with a direct, although not linear, relationship between the two. In terms of spatial variability, hotspots of reduced NO_2_ concentrations in the Northeast and California coincided perfectly with hotspots of reduced mobility. Vice versa, states where social distancing measures were minimal experienced the smallest reduction in NO_2_, e.g., Wyoming and North Dakota.

By contrast, the concentrations of PM_2.5_ did not decrease significantly, but rather increased slightly during the same period and even reached unprecedented high values at about a fifth of the sites. In addition, changes in PM_2.5_ concentrations were not correlated with changes in people’s mobility, neither spatially nor as aggregated statistics.

We propose that the different response to reduced people’s mobility between NO_2_ and PM_2.5_ could be explained by the fact that commercial vehicles (including delivery trucks, buses, trains), generally diesel fueled, remained more or less in circulation, while the use of passenger vehicles, which are primarily fueled by gasoline, decreased dramatically due to COVID-19. PM_2.5_ emissions are generally larger from diesel than from gasoline vehicles. In addition, other sources of PM_2.5_ emissions, like power plants and residential heating, did not decrease or even increased. Although our hypothesis is reasonable and consistent with the data that we have presented, the analysis conducted here is not a proof. We plan to verify this hypothesis in a subsequent study using a photochemical model coupled with a numerical weather prediction model, as described in Section 3.3, in conjunction with actual vehicle and air traffic data.

As far as we know, this is the first study to use ground monitoring stations to assess the effects of COVID-19 on air quality in the U.S. Satellite-derived NO_2_ column totals have been used in a few previous studies, but none looked at the correlation between the two types of NO_2_ measurements. Another innovation of this study is the use of mobility data, which are an excellent proxy for people’s actual behavior, as opposed to the state or county regulations, which may or may not be fully followed by people.

This analysis has a number of limitations. First of all, we paired mobility data and pollutant concentrations at the county level, thus we implicitly assumed that the measured concentration and county-average mobility were representative of the entire county. For large counties, especially those with also low population density, this assumption may not hold. The second implicit assumption of our pairing is that local mobility affects local pollution only and, vice versa, that local pollution is affected by local mobility only. In other words, we are neglecting the effects of transport and chemical reactions, which could cause either an increase or a decrease of pollution regardless of the local mobility change in the county of interest. For example, consider the case that the prevailing wind is such that a county is located downwind of an airport. If the airport was shutdown during the pandemic, that county would see a reduction of NO_2_ and PM_2.5_ concentrations even if no social distancing measures were in place. With a modeling approach, which we will pursue in the future, we will be able to properly capture the effect of transport. Another limitation is that we looked at people’s mobility as the only factor explaining NO_2_ concentration changes, whereas NO_2_ emissions changed also in response to business and school closures, air traffic reductions, among many others sources. Meteorological factors, such as colder or warmer than usual temperatures in certain locations, may also have affected air pollution indirectly, as people heated their homes more or less than usual in April 2020 with respect to previous years. Lastly, we focused on two pollutants only, NO_2_ and PM_2.5_, because of time constraints; future work will include other regulated compounds, such as ozone and carbon monoxide.
